# Tightening the requirements for species diagnoses would help integrate DNA-based descriptions in taxonomic practice

**DOI:** 10.1371/journal.pbio.3002251

**Published:** 2023-08-22

**Authors:** Frank E. Rheindt, Patrice Bouchard, Richard L. Pyle, Francisco Welter-Schultes, Erna Aescht, Shane T. Ahyong, Alberto Ballerio, Thierry Bourgoin, Luis M. P. Ceríaco, Dmitry Dmitriev, Neal Evenhuis, Mark J. Grygier, Mark S. Harvey, Maurice Kottelat, Nikita Kluge, Frank-T. Krell, Jun-ichi Kojima, Sven O. Kullander, Paulo Lucinda, Christopher H. C. Lyal, Cristina Luisa Scioscia, Daniel Whitmore, Douglas Yanega, Zhi-Qiang Zhang, Hong-Zhang Zhou, Thomas Pape

**Affiliations:** 1 National University of Singapore, Department of Biological Sciences, Singapore; 2 Canadian National Collection of Insects, Arachnids and Nematodes, Agriculture and Agri-Food Canada, Ottawa, Ontario, Canada; 3 Department of Natural Sciences, Bernice Pauahi Bishop Museum, Honolulu, Hawaii, United States of America; 4 Abteilung Evolution und Biodiversität der Tiere und Zoologisches Museum, Universität Göttingen, Göttingen, Germany; 5 Biology Centre of the Upper Austrian Museum, Linz, Austria; 6 Australian Museum, Sydney, Australia; 7 School of Biological, Earth and Environmental Sciences, University of New South Wales, Kensington, Australia; 8 Independent Researcher, Brescia, Italy; 9 Institut Systématique, Evolution, Biodiversité (ISYEB), MNHN-CNRS-Sorbonne Université-EPHE- Université des Antilles, Museum National d’Histoire Naturelle, Paris, France; 10 Departamento de Vertebrados, Museu Nacional, Universidade Federal do Rio de Janeiro, Rio de Janeiro, Brazil; 11 Illinois Natural History Survey, University of Illinois at Urbana-Champaign, Champaign, Illinois, United States of America; 12 National Museum of Marine Biology and Aquarium, Checheng, Taiwan; 13 Department of Terrestrial Zoology, Western Australian Museum, Welshpool DC, Australia; 14 Independent Researcher, Delémont, Switzerland; 15 Department of Entomology, Saint-Petersburg State University, Saint Petersburg, Russia; 16 Denver Museum of Nature and Science, Denver, Colorado, United States of America; 17 Natural History Laboratory, Faculty of Science, Ibaraki University, Mito, Japan; 18 Department of Zoology, Swedish Museum of Natural History, Stockholm, Sweden; 19 Laboratório de Ictiologia Sistemática, Universidade Federal do Tocantins, Tocantins, Brazil; 20 Department of Life Sciences, Natural History Museum, London, United Kingdom; 21 Arachnology Division, Museo Argentino de Ciencias Naturales ‘Bernardino Rivadavia’, Buenos Aires, Argentina; 22 Staatliches Museum für Naturkunde Stuttgart, Stuttgart, Germany; 23 Department of Entomology, University of California, Riverside, Riverside, California, United States of America; 24 Manaaki Whenua–Landcare Research, Auckland, New Zealand; 25 School of Biological Sciences, University of Auckland, Auckland, New Zealand; 26 Institute of Zoology, Chinese Academy of Sciences, Beijing, People’s Republic of China; 27 Zoological Museum, Natural History Museum of Denmark, Copenhagen, Denmark

## Abstract

Modern advances in DNA sequencing hold the promise of facilitating descriptions of new organisms at ever finer precision but have come with challenges as the major Codes of bionomenclature contain poorly defined requirements for species and subspecies diagnoses (henceforth, species diagnoses), which is particularly problematic for DNA-based taxonomy. We, the commissioners of the International Commission on Zoological Nomenclature, advocate a tightening of the definition of “species diagnosis” in future editions of Codes of bionomenclature, for example, through the introduction of requirements for specific information on the character states of differentiating traits in comparison with similar species. Such new provisions would enhance taxonomic standards and ensure that all diagnoses, including DNA-based ones, contain adequate taxonomic context. Our recommendations are intended to spur discussion among biologists, as broad community consensus is critical ahead of the implementation of new editions of the International Code of Zoological Nomenclature and other Codes of bionomenclature.

## Abstract 2

In this Essay, the International Commission on Zoological Nomenclature weighs in on current challenges in accommodating DNA data in taxonomic descriptions and outlines possible solutions, while soliciting community-wide discussion ahead of the next edition of the International Code on Zoological Nomenclature.

## The Codes of bionomenclature

In a series of influential publications in the 1750s, Carl Linnaeus established a strictly binomial (the botanical term) or binominal (the zoological term) naming system for organisms that has evolved through the centuries and become adopted almost universally by biologists across the world to serve as the foundation of modern biological nomenclature. Linnaeus’s [1] *Species plantarum* in 1753 serves as the starting point for botanical, mycological, and phycological nomenclature as codified in today’s International Code of Nomenclature for algae, fungi, and plants (ICNafp). Similarly, the 10th edition of Linnaeus’s [2] *Systema naturae* (1758) is the starting point for zoological nomenclature, which today is regulated by the International Code of Zoological Nomenclature (ICZN). A bacterial counterpart, the International Code of Nomenclature of Prokaryotes (ICNP), first came into effect in 1980.

The primary objective of these Codes is to promote stability and universality of the scientific names of organisms. The associated rules and regulations have become more detailed over the decades and reflect biologists’ need for effective communication while respecting freedom of taxonomic thought. The Codes are regularly updated to address issues that may cause nomenclatural instability and to adjust regulations to new developments in science and publishing. The ICNafp is reviewed by the Nomenclature Section of the International Botanical Congress every 6 years, most recently in Shenzhen, China, which led to the current Shenzhen Code [3]. The zoological Code, ICZN [4], is revised at uneven intervals by a committee appointed by the International Commission on Zoological Nomenclature. The most recent (fourth) edition of this Code was published in 1999 and took effect at the start of 2000. A fifth edition, now being drafted, will be published this decade following a 1-year period of public review and commentary. The prokaryotic counterpart, the ICNP [5], is similarly updated as needed, with the current edition having been revised in 2008, and the drafting of a new edition now in progress [6,7]. The community’s participation in updating these Codes is crucial for nomenclature to work efficiently. However, constructive public input requires a thorough analysis of the complex problems that need to be addressed, as unequivocal solutions to such problems are often elusive, and a solution to a problem regarding one set of rules may create its own problems with other rules.

Among the major recent changes in modern taxonomic practice is the increasing reliance on data harvested by means of modern technological advances, first and foremost DNA sequencing and bioimaging [8], but also other approaches such as metabolomics [9,10] and near-infrared spectrometry [11]. In order to better understand the challenges that the nomenclatural accommodation of such new sources of data might pose, we first examine existing areas of controversy and disagreement in how newly proposed species have been diagnosed or described.

### The requirement for a “diagnosis” or “description” in proposals for new names of species

All 3 major Codes of bionomenclature require that a new species name be accompanied by a statement providing the means whereby the species can be recognized and distinguished from other species (Box 1). Such statements are variously referred to as diagnoses, definitions, or descriptions, with varying opinions about what sets these words apart. For simplicity, herein, we use the word “diagnosis” to refer to such a statement of potentially distinguishing features.

**Box 1. The 3 Codes’ requirement for statements providing distinguishing features when describing new species or subspecies**

All 3 major Codes of bionomenclature contain stipulations that a new species or subspecies name be accompanied by a statement providing the means whereby the taxon can be recognized and distinguished from other taxa. Although roughly equivalent, these requirements are worded differently among the 3 major Codes.

The ***International Code of Zoological Nomenclature*** (ICZN; [4]) contains a requirement in its Article 13.1.1 that names described after 1930 “…be accompanied by a description or definition that states in words characters that are purported to differentiate the taxon…”

The ***International Code of Nomenclature for algae*, *fungi*, *and plants*** (ICNafp; [3]) states, in its Article 38.1, that a new name must “…be accompanied by a description or diagnosis of the taxon…”, with diagnosis defined in Article 38.2 as “…that which in the opinion of its author distinguishes the taxon from other taxa…”

The ***International Code of Nomenclature of Prokaryotes*** (ICNP; [5]) contains Rule 27, stipulating that for a new name to be validly published, “…the properties of the taxon being described must be given…” within the publication, while Rule 28a states that proposals to revive names proposed prior to 1980 “…must contain a brief diagnosis, i.e., a statement or list of those features that led the author to conclude that the proposed taxon is sufficiently different from other recognized taxa to justify its revival…”

While all 3 Codes have roughly equivalent requirements for statements providing distinguishing features (or “diagnoses”), the wording in each Code is sufficiently vague to make it impossible to ascertain whether diagnoses must be contrastive and/or state-specific (Box 2).

Outwardly simple, this requirement for a diagnosis has led to ongoing controversy over what exactly constitutes a Code-compliant way of presenting distinguishing features for any particular newly named species. Disagreement has mostly centred on 2 distinct but intersecting concepts, i.e., whether a diagnosis must be (1) contrastive (Box 2) and/or (2) specific with regard to character states (henceforth, “state-specific”) (Box 2). The definitions of “diagnosis” or comparable terms across the 3 Codes of bionomenclature are too vague to provide precise guidance on this question (Boxes 1 and 2). However, at a minimum, there is general agreement in the biological community that diagnoses that are both contrastive and state-specific constitute the gold standard.

**Box 2. Contrastiveness and state-specificity—Two ideal properties of diagnoses in species and subspecies descriptions**

All 3 Codes of bionomenclature require that a species or subspecies description be accompanied by a statement providing distinguishing features (here termed “diagnosis”; Box 1). But the loose definitions of “diagnosis” or comparable terms across the 3 Codes have generated controversy among taxonomists. Disagreement has centred mostly on 2 distinct but intersecting concepts, i.e., whether a diagnosis must be (1) contrastive and/or (2) specific with regard to character states (i.e., “state-specific”).

**Contrastive diagnoses**

A contrastive diagnosis presents distinguishing characters or character states in direct comparison to at least one other species, for example:

…Diagnosis: New Species X differs in leg colour from Species Y…

…Diagnosis: New Species X has green legs, while Species Y has red legs…

Most taxonomists would also consider a statement pointing to a unique character state as contrastive, even though the species of comparison is not explicitly named. For example:

…Diagnosis: The green leg colour of new Species X is unique among members of its genus…

Not all diagnoses are contrastive, as authors may content themselves with pointing out the leg colour of the new species without reference to comparable species.

**State-specific diagnoses**

A state-specific diagnosis is one in which an author not only presents a distinguishing character but also specifies its character state. For example:

…Diagnosis: The new species has green legs…

Some contrastive diagnoses mention only the character without specifying its state and are therefore non-state-specific, as in:

…Diagnosis: New Species X differs from Species Y in its leg colour…

A diagnosis may fail to be both state-specific and contrastive at the same time, as in…

…Diagnosis: The new species differs in leg colour…

…without providing the actual colour nor specifying the species from which it differs in this respect.

The definitions of “diagnosis” or comparable terms across the 3 Codes of bionomenclature are too vague to provide specific guidance on whether diagnoses must be contrastive and/or state-specific. For example, Example 4 of ICNafp Article 38.2 [3] seems to prohibit diagnoses that are not state-specific, while the definition of “diagnosis” in that very same article and in the ICNafp glossary would allow them.

At a minimum, there has been a general tacit agreement among many biologists that diagnoses that are both contrastive and state-specific constitute the gold standard. More public debate about future requirements for state-specificity and contrastiveness in diagnoses is urgently needed and encouraged.

### How have taxonomists dealt with diagnoses that fail to be contrastive and/or state-specific?

Taxonomy is replete with species descriptions that fail to be contrastive or state-specific, frustrating later biologists’ efforts to recognize species without having to consult the physical name-bearing type. Providing non-contrastive diagnoses is considered poor taxonomic practice and is actively discouraged in wide quarters of the biological community [12] but has been overwhelmingly tolerated by users and interpreters of the 3 major Codes of bionomenclature if the context makes it clear that a given set of characters is meant to differentiate (Box 2). Regarding diagnoses that fail to be state-specific, the situation is more muddled. The zoological community has often, but not always, considered such diagnoses unacceptable and rejected the corresponding names as unavailable, although a literal reading of the definition of key words in relevant Code sections is equivocal (Boxes 1 and 2). These deficiencies of the current Codes are being addressed by the various nomenclatural bodies at present.

While the problem of non-contrastive and/or non-state-specific diagnoses has long been recognized, the public debate about this topic has been rather muted, indicating that most biologists have not felt any great urgency to address it. One reason for this stance may lie in the historical trend towards increasing precision in taxonomic practice. Whereas published descriptions in the first 2 centuries after Linnaeus customarily contained a bare minimum of information, often confined to simple minimalistic descriptors, most modern descriptions of new species typically include detailed diagnoses, which list many different characters. Given that modern diagnoses are—on average—so much richer than those of past centuries, the occasional (or even frequent) lack of contrastiveness or state-specificity is not necessarily as limiting as it would have been in the 1800s.

In summary, against the backdrop of the imprecise language in Code definitions regarding what does and does not constitute a diagnosis, the taxonomic community has converged on consensus practices, and our nomenclatural system has thrived without major threats to its stability. However, following the advent of the genomic revolution, it is important to ask whether the generally vague definitions of diagnoses, which are not overly problematic at this point in time, will continue to remain effective in preventing major rifts in the face of DNA-based species descriptions and other potential new practices.

### The DNA revolution in taxonomy

Immense technological advancements over the last few decades have facilitated the compilation of datasets of unprecedented volume that biologists can use for taxonomic purposes. These new data sources are manifold and range from biochemical pheromone characterizations to morphological descriptions via bioimaging but are currently dominated by DNA sequencing, on which we will focus here. The biological community has come a long way from the single-marker sequencing that originated in the early PCR revolution of the 1980s to today’s genome sequencing, which is based on an entirely new generation of technology.

The availability of such large quantities of data has accelerated taxonomic progress across many groups of organisms. It has revolutionized and sometimes overturned our understanding of even very basic biological concepts and allowed for an ever-finer delimitation of species by breaching the frontiers of morphological insights [13]. However, while DNA datasets have provided the impetus for myriads of new species descriptions since the 1980s, only a modest number of nominal species have been formally diagnosed primarily based on molecular data (<1,000 species of animals according to literature survey; [14–19]). In the vast majority of modern descriptions associated with DNA data, tree diagrams or divergence estimates based on DNA alignments have indicated the distinctness of a species, but authors have relied on morphological or behavioral traits for the actual diagnoses.

### Sequence-based diagnoses on the rise

The addition of DNA data to our taxonomic arsenal has bolstered the modern trend of integrative species descriptions, which is likely to continue with further technological advancements. Even so, there are multiple reasons to believe that diagnoses based purely on molecular sequence data will become increasingly commonplace in future taxonomic practice and may replace morphology-based diagnoses almost entirely in certain groups of organisms. Some of these reasons hinge on trends in society: Decades-long shifts in the global funding landscape have led to precipitous declines in taxonomic infrastructure and expertise [20,21] against the backdrop of a continual rise in DNA-related research. Independently, DNA-sequencing capabilities have expanded exponentially [22–24]. Whereas an average PhD student in the early 1990s would take a year to produce an approximately 1,000-base dataset for approximately 50 individuals, the same student today could produce entire vertebrate genomes (>1 billion bases) for the same number of individuals in the same timeframe, which translates into 3 to 4 orders of magnitude more data.

Other reasons for a likely future increase in purely sequence-based diagnoses are related to our growing appreciation of the magnitude of cryptic diversification. Taking flies (Diptera) as an example, a nearly inexhaustible volume of species remain to be described on morphological grounds, but taxonomists additionally realize that multiple cryptic species may be embedded in almost every one that is recognized through morphology [25–28]. In yet other groups of organisms (e.g., fungi and various unicellular organisms) new species have sometimes been identified exclusively based on environmental DNA samples, precluding a description by any means other than a DNA sequence [29,30]. DNA barcoding, described in further detail below, has been a simple, cheap, and convenient way to rapidly separate numerous novel cryptic insect species, providing taxonomists with a starting point for morphological inquiry.

### The taxonomic impediment and cryptic diversity

Even by the most conservative estimates of total global biodiversity, the vast majority of Earth’s species (under any definition of the term “species”) remain undescribed [31]. An increasing body of research shows that the species count in many insect groups may, on average, increase by an order of magnitude when cryptic species are taken into account [13,16,17]. This suggests that even our vague current estimates of undescribed diversity may be too low.

In assemblages of taxonomically cryptic organisms, species names associated with a DNA barcode tend to have greater taxonomic utility in many contexts than those without a barcode. Given nomenclature’s long history, many scientific names are based on old name-bearing types, which often do not readily lend themselves to DNA analysis, or in some cases are even lost, rendering such names *nomina dubia* once it is recognized that they may in fact represent any of multiple cryptic species. As DNA-based advances in taxonomic insight result in potentially thousands of insect names becoming *nomina dubia*, it is unsurprising that some researchers have called for DNA barcodes to become a mandatory component of future descriptions and diagnoses [32,33]. These petitions have been countered by some quarters of the traditional taxonomic field as impracticable for many organisms (especially fossils) and as discriminatory against researchers who lack molecular resources and expertise [34–37].

On the other extreme, some biologists have gone further in establishing new codes and practices allowing for DNA sequences not only to feature within nomenclatural diagnoses but also to function as the actual name-bearing type, in the same way as collection specimens conventionally serve as type specimens [38–40]. These new movements largely focus on groups of organisms that are notoriously challenging to collect, fix, deposit, or keep, such as protists and certain fungi [38–40]. Their actions and practices are considered outside of the remit of the 3 long-established Codes of bionomenclature (ICZN, ICNafp, and INCP), and petitions to adopt DNA sequences as types are currently not being considered at least by the framers of the 2 Codes that cater to many macroorganisms (ICZN and ICNafp).

### DNA barcode-based diagnoses in practice

The integration of molecular data into taxonomic descriptions has taken multiple forms, each with its own nomenclatural problems (see [41]). Some authors, for example, provide descriptions in which a DNA barcode constitutes the core element of the diagnosis [16,17]. Barcodes overwhelmingly comprise DNA sequences of mitochondrial genes and are usually anywhere between 500 and 1,200 bases (i.e., the letters A, C, T, and G) in length, with some variation. Diagnoses based solely on DNA barcode sequences, without explicit indication of which positions in the sequence differ from those of other species, are essentially non-contrastive, i.e., they are akin to statements such as “…the new species has green legs…” that fail to provide a comparison to the leg colour of other species (Box 2). As was mentioned above, such non-contrastive diagnoses are widely criticized, yet there is also a long-standing tradition to accept such names if a good-faith attempt on part of the authors to provide a diagnosis is recognizable. Some (but not all) barcode diagnoses contain a statement that the presented barcode is unique among all known members of the genus, which would confer at least an arguable degree of contrastiveness upon them. At the same time, many taxonomic practitioners still regard such diagnoses as problematic because the investigative burden on the user can be much greater than in most morphological diagnoses.

In other cases, authors have diagnosed species on the basis of divergence or DNA distance values, leading to statements such as “…new Species A exhibits a 3.5% uncorrected divergence from Species B in the COI barcoding gene…”. While contrastive between species, this type of diagnosis provides no character states (Box 2). In other words, such a statement is akin to saying that the leg morphology of 2 species somehow differs by 3.5%, with no indication about which specific leg traits are being referred to. There has been a long-standing tacit tradition in taxonomy not to accept diagnoses that fail to be state-specific, quite unlike the tolerance that has generally been extended to non-contrastive diagnoses.

Unfortunately, the wording of current Code editions is equivocal regarding the permissibility of descriptions that fail to be contrastive or state-specific (Boxes 1 and 2), and it is imperative to update current Codes to be clear about which forms of diagnoses—regardless of whether molecular or morphological—are Code compliant.

Ideal incarnations of barcode-based diagnoses are both contrastive and explicit with regard to character states at the same time. For example, the ideal presentation of a barcode in a diagnosis should be accompanied by statements regarding specific unique positions within the DNA sequence. An example of this would be “…the new species differs from all other species of the genus by two synapomorphies in the COI gene: at base 49 there is a substitution to T; and at base 514 there is a substitution to C…”. When diagnostic positions are tagged relative to their position in the reference sequence of a commonly used model species (e.g., *Homo sapiens*, *Drosophila melanogaster*, *Arabidopsis thaliana*) rather than in an alignment-specific way, such descriptions adhere to the gold standard of how diagnoses should be framed (Box 2) and are likely to remain immune to concerns by critics. Although currently still uncommon, such barcode diagnoses can already be found in the literature (e.g., [42]), and an encouraging volume of new software has been published to automate and simplify such diagnoses [43–48].

### The dangers and advantages of barcode-based diagnoses

Critics of barcode-based diagnoses deplore the fact that the mere presentation of a string of letters representing nucleotides of a DNA sequence puts an immense burden on the user to isolate few distinctive elements from an avalanche of nondiagnostic background noise. Such diagnoses are especially intimidating to taxonomists who lack molecular training and struggle to make sense of such data. Proponents of barcode-based diagnoses, on the other hand, usually offer 3 lines of defense in favor of their approach: (1) DNA barcodes are mere strings of approximately 500 to 1,200 letters; once produced, they do not require the use of any dedicated technology to read, and they are as straightforward to analyse as any other sequence of coded biological character states; (2) species descriptions that contain barcode-based diagnoses usually also present more intuitive information on the true extent of divergence and distinctness of a new species outside of the diagnosis, either in the form of tree diagrams or divergence values; and (3) for many cryptic species, the barcode differences are all we have in the absence of diagnostic morphological traits.

The inclusion of nondifferential regions of a DNA sequence within a diagnosis is not problematic in itself: Purely morphological diagnoses also often include character states that do not differ from those of explicitly compared species. Such additional information on character state values—regardless of whether they are molecular or morphological—serves to further differentiate proposed new species from all other known species, including those not explicitly compared in the original description.

### Genome-scale data in nomenclature

With the development of high-throughput sequencing platforms in the 2000s, some biologists have moved on from the era of single-marker sequencing (e.g., DNA barcodes) to one in which entire genomes can be harvested. In prokaryotes, nomenclature on the basis of whole genomes is already a reality, driven by the relatively small genome size of these organisms and the fact that prokaryotes often do not lend themselves to classical typification, although a substantial part of this new DNA-based nomenclature in prokaryotes and some fungi is conducted outside the traditional domain of the ICNP [38–40,49]. Soon, we may see the first diagnosis of a eukaryote based exclusively on a full genome. This prospect is both exciting and fraught, because, while full genomes offer so much more leeway in providing diagnostic characters, the way such diagnoses are framed may range from poor (e.g., non-contrastive and non-state-specific) to extremely detailed.

This leads to the question of whether a diagnosis that simply provides a reference to an online repository of billions of base pairs constituting an entire genome would comply with the requirements of the various Codes, and how these requirements should be refined to better serve our community. The same applies to diagnoses linking to DNA sequences based on “ultraconserved elements” (UCEs) or RADseq datasets of millions of base pairs.

### Problems with potential future diagnoses based solely on references to genome sequence archives

Sequence-based diagnoses pose a certain burden on the user in that the characters cannot easily be assessed without access to a computer, reliable connectivity, and a modicum of analytical knowledge. While morphological diagnoses may sometimes require even more expensive equipment (e.g., electron microscopes), their resulting measurements are usually easier to grasp for a reader than a sequence of DNA letters. At the same time, most DNA-based diagnoses are (or should be) accompanied by additional context, such as divergence figures between closely related species or tree diagrams, translating the information content of the diagnostic DNA bases into easily digestible information.

While concerns raised about sequence-based diagnoses can often be addressed in datasets based on simple DNA barcodes spanning approximately 1,000 bases, they are compounded in diagnoses exclusively based on genome-wide DNA sequences, which can extend to tens of billions of base pairs and therefore exceed DNA barcodes in length by up to 7 orders of magnitude. It is widely overlooked that the difference between a barcode-based diagnosis and one based on whole genomes is not merely a matter of scale. Barcode sequences of a group of target species are easily alignable and can be subjected to standard divergence analysis and tree-building algorithms within a matter of minutes. In fact, in the absence of a computer, approximately 800-base barcodes can even be compared manually with paper and pen if absolutely needed. In contrast, across whole genomes, regions of taxonomic utility occupy a minute percentage of the entire chromosomal space, sometimes as little as approximately 5% (e.g., [50]), while the remainder consists of stretches of up to tens of millions of base pairs of unalignable hypervariable or highly repetitive regions, mostly of unknown functionality and hence widely termed “junk DNA” [51,52]. Aligning genomes across various target species will always remain challenging regardless of technological advances, and no two analyses will ever be the same, as slight adjustments to analytical parameters will lead to the inclusion or exclusion of vast tracts of DNA data. Asking a reader to pick and choose a small minority of useful traits from among billions (even by using a computer program) would exceed by orders of magnitude anything that has previously been asked of consumers of the taxonomic literature. Therefore, diagnoses that simply link to a genome sequence archive without further context would seriously challenge our current model of nomenclaturally permissible species descriptions, which places the burden of identifying diagnostic character states on the author, not the user.

Ultimately, the presentation of an entire genome sequence as a non-contrastive diagnosis could be considered the molecular analog of presenting a 3D microtomography image of an entire holotype specimen as one single diagnostic character and leaving it to readers to find relevant traits. Would the biological community be comfortable allowing a photograph of an entire organism to count as a presentation of “distinguishing character states” without any additional words? For animals, this is unlikely, because the ICZN revoked this option for species descriptions after 1930. Would we be comfortable with the molecular analog of such a scenario? These are the questions the biological community must contemplate as stakeholders are starting to draft new editions of the 3 nomenclatural Codes that will come into force during the crucial transition period to a genomic future.

### Our recommendation for future editions of Codes of bionomenclature

Updates and/or new editions of all the Codes of bionomenclature are impending, with opportunities for the scientific community to express its concerns and predilections while offering suggestions to improve the Codes. Our 3 major Codes do not constitute prescriptive dictates but are conceived and framed by specific stakeholder bodies based directly on feedback from the biological community. As we are entering a new era in taxonomy, open discussion of issues and their potential remedies is of paramount importance if the framers of the Codes are to properly gauge public preferences and compose rules and regulations that continue to inspire near-universal respect and acceptance.

We advocate an explicit requirement for state-specific and contrastive diagnoses. If widely supported by biologists, such a new requirement could—in the case of the ICZN, for example—be incorporated into the impending fifth edition of the zoological Code, stipulating that future species descriptions must contain a diagnosis that clearly provides information on at least one specific character and its character state in which the new species differs from closely related species.

We anticipate that such a tightening of Code rules would be welcomed by wide quarters of the community, as it would substantially increase the general quality of taxonomic diagnoses. To be clear, such new requirements would not stipulate certain standards of taxonomic quality; i.e., we are not advocating requirements of a minimum number of characters or species to be compared, as this would impinge on taxonomic freedom, given that the nature of ideal comparisons differs among groups of organisms.

Such new requirements would still allow for a range of formats for DNA-based diagnoses, including in new organisms for which unique nonmolecular characters are unknown. The gold standard for such DNA-based diagnoses, which can already be found in the literature (e.g., [42]), would ideally encompass a presentation of unique loci or locus combinations, replete with the diagnostic nucleotides found at these loci. At the same time, this new stringent requirement would effectively rein in potential excesses such as the linking of diagnoses to entire genome archives without further context.

We feel that a continuation of the broadly permissive approach of the current Codes, allowing for inclusivity of diagnoses that are not adequately state-specific and contrastive, would come with the risk that, by the 2030s, the biological field could be overrun with DNA-based “nondiagnoses” solely based on references to massive sequence archives without taxonomic context or interpretation. In the absence of rules against this practice, authors may publish the names of new nominal species based on such archives in good faith without being aware of potential downstream problems. Such a development may ultimately threaten the stability of bionomenclature if a sufficient number of biologists feel that their field has been inundated with problem-laden names, prompting them to adopt alternative naming systems.

Biologists all over the world should weigh-in now across all the relevant forums, providing the framers of future editions of the Codes with the strongest possible foundation for revising the criteria for Code compliance in the naming of species.

## Abbreviations

ICNafp: International Code of Nomenclature for algae, fungi, and plants
ICNP: International Code of Nomenclature of Prokaryotes
ICZN: International Code of Zoological Nomenclature
UCE: ultraconserved element
